# Trends of Human Plague, Madagascar, 1998–2016

**DOI:** 10.3201/eid2502.171974

**Published:** 2019-02

**Authors:** Voahangy Andrianaivoarimanana, Patrice Piola, David M. Wagner, Fanjasoa Rakotomanana, Viviane Maheriniaina, Samuel Andrianalimanana, Suzanne Chanteau, Lila Rahalison, Maherisoa Ratsitorahina, Minoarisoa Rajerison

**Affiliations:** Institut Pasteur, Antananarivo, Madagascar (V. Andrianaivoarimanana, P. Piola, F. Rakotomanana, S. Chanteau, L. Rahalison, M. Rajerison);; Northern Arizona University, Flagstaff, Arizona, USA (D.M. Wagner);; Ministry of Public Health, Antananarivo (V. Maheriniaina, S. Andrianalimanana, M. Ratsitorahina)

**Keywords:** Yersinia pestis, bacteria, plague, human plague, bubonic plague, pneumonic plague, trends, case-fatality rate, rats, fleas, zoonoses, Madagascar

## Abstract

Madagascar is more seriously affected by plague, a zoonosis caused by *Yersinia pestis*, than any other country. The Plague National Control Program was established in 1993 and includes human surveillance. During 1998–2016, a total of 13,234 suspected cases were recorded, mainly from the central highlands; 27% were confirmed cases, and 17% were presumptive cases. Patients with bubonic plague (median age 13 years) represented 93% of confirmed and presumptive cases, and patients with pneumonic plague (median age 29 years) represented 7%. Deaths were associated with delay of consultation, pneumonic form, contact with other cases, occurrence after 2009, and not reporting dead rats. A seasonal pattern was observed with recrudescence during September–March. Annual cases peaked in 2004 and decreased to the lowest incidence in 2016. This overall reduction occurred primarily for suspected cases and might be caused by improved adherence to case criteria during widespread implementation of the F1 rapid diagnostic test in 2002.

Plague, caused by the bacterium *Yersinia pestis*, produced some of the most devastating epidemics in human history; it is endemic to regions of Asia, the Americas, and Africa. Africa accounts for >90% of global human plague cases, and most cases are reported from Madagascar and the Democratic Republic of the Congo (*1*). 

Plague was introduced to Madagascar in 1898 at the port of Toamasina and reached the capital (Antananarivo) in 1921. It is endemic to the central and northern highlands (elevation >800 m). The main reservoir is the black rat (*Rattus rattus*), and 2 main flea species (*Xenopsylla cheopis*, a cosmopolitan species, and *Synopsyllus fonquerniei*, an endemic species) are involved in transmission. *S. fonquerniei* fleas show a greater transmission efficiency (*2**,**3*). Restriction of plague primarily to the highlands is probably caused by the absence of *S. fonquerniei* fleas at elevations <800 m (*4*).

Human plague has 2 primary clinical forms, bubonic and pneumonic (*5*). Bubonic plague (BP), the predominant form, is acquired by fleabite. Pneumonic plague (PP), which is less common, might arise from BP by hematogenous spread to lungs or inhalation of aerosols during human-to-human transmission. Without treatment, case-fatality rates (CFRs) are 40%–70% for BP and 100% for PP (*2*,*6*,*7*). 

In Madagascar, per the Plague National Control Program (PNCP), reporting of suspected human cases by health centers is mandatory. Confirmation is performed by the Central Laboratory for Plague of the Malagasy Ministry of Health, which is hosted at the Plague Unit of the Institut Pasteur (Antananarivo, Madagascar). We report epidemiologic data and trends for human plague in Madagascar over 19 years of surveillance, analyze risk factors associated with plague complications, and evaluate the specificity and sensitivity of the rapid diagnostic test for *Y. pestis* antigen fraction 1 detection (F1RDT) (*8*) after 15 years of use.

## Data Analysis

We analyzed data for human plague surveillance activities (Appendix) by using Stata 14 (StataCorp LLC, https://www.stata.com). We also analyzed epidemiologic trends of calculated suspected, presumptive, and confirmed cases. Cases were considered confirmed if *Y. pestis* was isolated by culture or mouse inoculation, presumptive if positive results were obtained by F1RDT or microscopy (i.e., gram-negative coccobacilli showing bipolar staining) without isolation of *Y. pestis*, or suspected if no samples were available for testing or all test results were negative (*9*).

**Figure 1:**
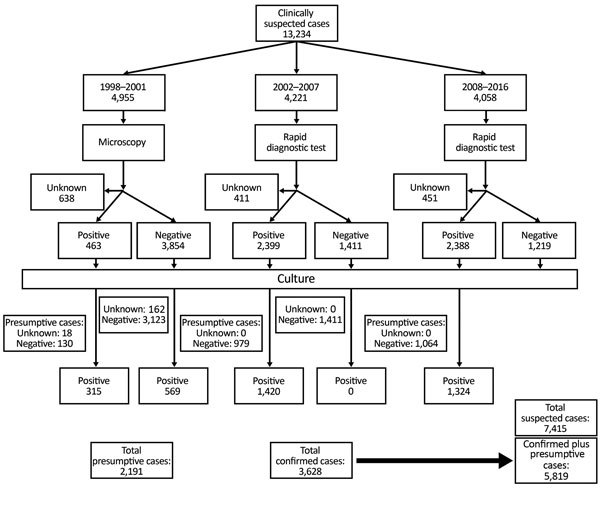
Diagnostic flowchart for suspected cases of plague, Madagascar, 1998–2016.

Changes in screening tests resulted in different case definitions: before 2002, the screening test was microscopy, but during 2002–2008, F1RDT replaced microscopy. We estimated performance of microscopy (1998–2001) and F1RDT (2002–2007) by measuring sensitivity and specificity compared with bacteriological culture; from 2008 on, only positive F1RDT results at the Central Laboratory for Plague were confirmed by culture. Cases for which samples were not available for testing are listed as unknown (Figure 1) and were excluded from sensitivity and specificity analyses. 

A retrospective case–control study identified death risk factors for confirmed plus presumptive cases. Case-patients were persons who died shortly before or after diagnosis of plague; controls were persons who survived. Potential factors examined included delay to consultation after symptom onset (categorized as 0, 1, or >2 days); clinical form (BP or PP); time period (before 2009 or from 2009 on); contact with another confirmed plus presumptive case; reporting presence of dead rats (yes/no); age (<18 years of age); sex; recent travel in a plague-endemic focus area (yes/no); month of plague reporting; and mean elevation of district residence. Clinical form was defined on the basis of when the case was initially examined. Thus, PP cases included persons with primary PP and secondary PP arising from BP. Recent travel was defined as traveling in a plague-endemic focus area within 10 days before disease onset. Elevation was included because it has been described as a risk factor for death from pneumonia (*10*).

To assess death risk factors, we also analyzed all variables associated with death in univariate analysis at the p<0.2 level in a multivariate fashion by using logistic regression. Only confirmed plus presumptive cases were used for additional analyses, including estimation of plague incidence, geographic localization of outbreaks, description of infection clusters (groups of >2 confirmed case-patients or presumptive case-patients who reported physical contact, close proximity, or other possible infection links with each other), and environmental risk factors of plague endemicity.

## General Trends

**Figure 2:**
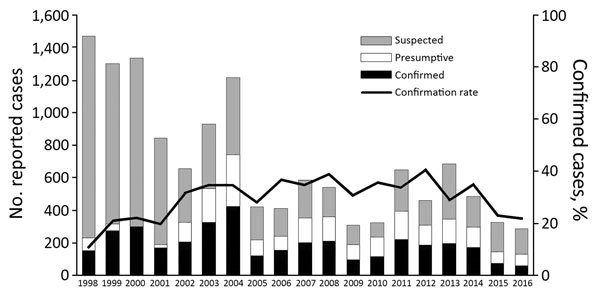
Classification of reported plague cases and confirmation rate, Madagascar, 1998–2016.

During January 1, 1998–December 31, 2016, a total of 13,234 clinically suspected plague cases were reported to the PNCP. Of these cases, 3,628 (27%) were classified as confirmed and 2,191 (17%) as presumptive (Figures 1, 2); the confirmation rate improved gradually over time (Figure 2). For the 5,819 confirmed plus presumptive cases, PP represented 7% (409) and BP 88% (5,132). The remaining 278 (5%) were categorized as neither PP or BP; because the reporting form only included information on symptoms of BP and PP and not other forms of plague, these other cases were not further differentiated in terms of clinical manifestation. 

Yearly proportion of PP for confirmed plus presumptive cases ranged from 0.3% (1/308) during 2002 to 14.7% (20/136) during 2015; there was a statistically significant increase in proportion of PP during 1998–2016 (slope 0.0055; p<0.0001). We found no significant difference in proportion of PP and BP between suspected cases (91.7% BP) and confirmed plus presumptive cases (92.6% BP; p = 0.08).

**Figure 3:**
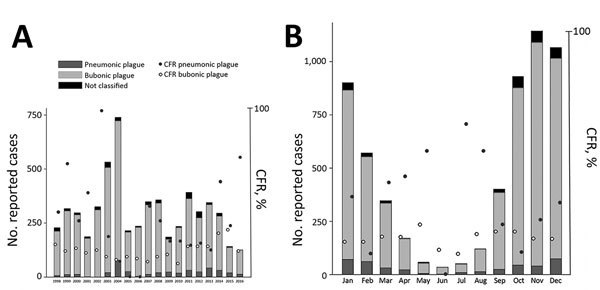
Yearly (A) and monthly (B) distributions of bubonic and pneumonic plague and their CFR, Madagascar, 1998–2016. CFR, case-fatality rate.

The CFR was 18% (1,057/5,819) for confirmed plus presumptive case-patients and significantly higher for patients with PP (25%, 102/409) than for patients with BP (15%, 747/5,132; p<0.001). The yearly CFR for BP ranged from 8.2% (2010) to 28.4% (2015) but was much wider for PP (0% during 2005–2006, 100% during 2002) (Figure 3), likely because of relatively few PP cases. Annual confirmed plus presumptive cases peaked in 2004 (n = 739; 9% were reported from Antananarivo-Renivohitra) and were lowest in 2016 (n = 126). Monthly distribution of cases showed an increase in September, a peak in November, a progressive decrease until April, and then few cases until August (Figure 3).

## Performance of Microscopy and F1RDT

Using culture as a reference, we determined that microscopy had a sensitivity of 35.6% (315/884; samples from 1998–2001) and a specificity of 96% (3,123/3,253). F1RDT sensitivity was 100% (1,411/1,411; samples from 2002–2007) and specificity was 59% (1,420/2,399); F1RDT specificity was higher for BP (60.5%; samples from 2002–2007) than for PP (44.7%; p<0.001). Because of 100% sensitivity of F1RDT (Table 1), only positive F1RDT results were confirmed by culture since 2008. Thus, it was not possible to examine specificity of F1RDT during 2008–2016.

**Table 1:** Sensitivity and specificity of microscopy and F1RDT for the diagnosis of human plague cases, Madagascar*

Clinical form	Microscopy, 1998–2001			F1RDT, 2002–2008	
	Sensitivity	Specificity		Sensitivity	Specificity
Bubonic	34% (31%–38%)	96% (95%–97%)		100% (99%–100%)	60% (58%–62%)
Pneumonic	53% (34%–71%)	94% (84%–98%)		100% (90%–100%)	45% (37%–52%)
Total	35% (32%–39%)	96% (95%–97%)		100% (99%–100%)	59% (57%–61%)

*Values in parentheses are ranges. Bacteriological culture was used as a reference test. F1RDT, *Yersinia pestis* antigen fraction 1 rapid diagnostic test.

## Risk Factors Associated with Plague

Sociodemographic and epidemiologic characteristics did not vary between confirmed plus presumptive cases and suspected cases (Table 2). For confirmed plus presumptive case-patients, we found a significant age difference between PP cases (median 29 years) and BP cases (median 13 years; p<0.001). In addition, reporting dead rats was higher for BP (18.6%) than PP (9.3%) case-patients (p<0.001), and the proportion of recent trips (i.e., trip in a plague-endemic area for 10 days before reporting) was almost 2-fold greater for PP (13.7%) than for BP (6.9%) case-patients (p<0.001).

**Table 2:** Sociodemographic and epidemiologic characteristics of persons with confirmed, presumptive, and suspected plague cases, Madagascar, 1998–2016*

Characteristic	Bubonic plague case status, no. patients			Pneumonic plague case status, no. patients	
	Suspected, n = 6,454	Confirmed and presumptive, n = 5,132		Suspected, n = 579	Confirmed and presumptive, n = 409
Median age, y (IQR)	11 (6–20)	13 (8–24)		26 (17–40)	29 (20–42)
Sex ratio (M:F)	1.44 (3,803:2,639)	1.38 (2,972:2,157)		1.08 (300:278)	1.28 (230:179)
Presence of rats, %	15.1	18.6		11.3	9.3
Recent trip, %	6.4	6.9		7.0	13.6
Mean (SE) days to care	2.0 (0.05)	1.7 (0.03)		2.61 (0.15)	2.21 (0.18)
Median elevation, m (IQR)	1,262 (1,111–1,384)	1,275 (1,063–1,409)		1,228 (921–1,333)	1,284 (1,111–1,355)
Contact with other plague cases, %	7	11		21	23

*IQR, interquartile range.

**Figure 4:**
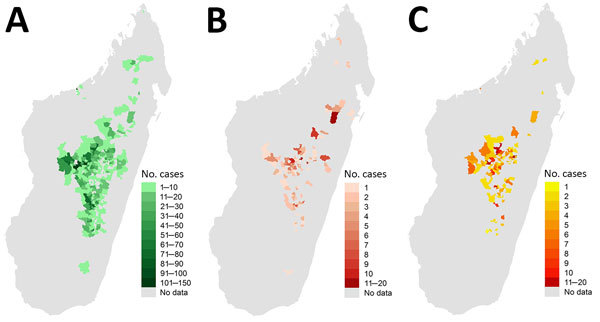
Geographic distributions of bubonic plague (A), pneumonic plague (B), and infection clusters (C), Madagascar, 1998–2016.

Confirmed plus presumptive cases were concentrated mostly in the central highlands (Figure 4). Most BP cases occurred in the west–northwest region, whereas hotspots of PP were found in the northeastern central highlands. Median elevation of district of residence was similar for BP and PP case-patients (Table 2), within 1,200 m–1,300 m. In the 415 communes where plague cases were reported, yearly incidence estimates from census population data were 0.03–63 cases/100,000 persons/year (median 1.4 cases/100,000 persons/year, interquartile range 0.7–3.2 cases/100,000 persons/year). Of 5,819 confirmed plus presumptive cases, 473 (8.1%) were in an infection cluster; many infection clusters occurred in the middle of the central highlands (Figure 4). There were 211 infection clusters: 76% involved 2 contacts (of which 10% involved PP), 18% involved 3 contacts (11% PP), 4% involved 4 contacts (16% PP), and 1% involved 5 contacts (10% PP).

We found by univariate analysis that death was associated more with PP (odds ratio [OR] 2.0, 95% CI 1.57–2.53) than with BP. For PP case-patients, death was associated with delayed consultations (OR 2.7, 95% CI 1.70–4.37), occurrence after 2009 (OR 1.60, 95% CI 1.02–2.52), and age group (OR  0.50, 95% CI 0.29–0.85). For BP case-patients, death was associated with delayed consultations (OR 3.44, 95% CI 2.92–4.04), contact with another case-patient (OR 1.86, 95% CI 1.45–2.37), occurrence after 2009 (OR 1.56, 95% CI 1.33–1.83), and not reporting dead rats (OR 0.67, 95% CI 0.54–0.85).

When we combined BP and PP case-patients (Table 3), we found that death was associated with age, sex, and recent travel. However, month and mean elevation of district of residence were not associated with death, except for the month of August. In the multivariate model (Table 3), we found that the remaining factors with an association after adjustments were delayed consultation (from OR 3.2 at day 2 up to OR 5.0 at day 4), pneumonic plague (OR 1.6), contact with another plague case (OR 1.9), year 2009 or after (OR 1.6), and not reporting dead rats (OR  0.7) (all p values <0.001). Persons >35 years of age had a moderately higher risk for death than did persons <35 years of age (OR 1.25, p = 0.04).

**Table 3:** Risk factors for death from plague, Madagascar, 1998–2016*

Factor	No. patients	Univariate analysis			Multivariate analysis	
		OR (95% CI)	p value		OR (95% CI)	p value
Delay to seek healthcare, d						
0 (same day)	1,142	1			1	
1	2,426	0.91 (0.73–1.14)	0.42			
2	1,059	2.51 (2.00–3.16)	<0.0001		3.24 (2.46–4.28)	<0.0001
3	504	3.94 (3.01–5.14)	<0.0001		4.93 (3.62–6.70)	<0.0001
4	288	3.99 (2.92–5.44)	<0.0001		4.98 (3.48–7.11)	<0.0001
>5	385	2.20 (1.63–2.97)	<0.0001		2.94 (2.08–4.14)	<0.0001
Clinical form of plague						
Bubonic	5,114	1			1	
Pneumonic	401	2.00 (1.57–2.53)	<0.0001		1.58 (1.21–2.06)	<0.0001
Time period						
Until 2009	3,977	1			1	
After 2009	1,843	1.68 (1.46–1.93)	<0.0001		1.58 (1.34–1.86)	<0.0001
Contact with a plague case						
No	5,275	1			1	
Yes	486	1.92 (1.55–2.38)	<0.0001		1.90 (1.47–2.41)	<0.0001
Reporting dead rats						
No	4,544	1			1	
Yes	971	0.63 (0.50–0.77)	<0.0001		0.66 (0.52–0.82)	0.001
Age, y						
<5	589	1			1†	
5–18	2,964	0.88 (0.70–1.11)	0.28			
19–35	1,383	0.93 (0.73–1.20)	0.58			
36–54	614	1.42 (1.08–1.88)	0.01		1.25 (1.01–1.56) 0.04†	
>55	156	1.38 (0.91–2.11)	0.12			
Sex					ND	ND
F	2,438	1			ND	ND
M	3,319	0.86 (0.75–0.98)	0.026		ND	ND
Recent travel					ND	ND
No	5,214	1			ND	ND
Yes	422	1.37 (1.07–1.75)	0.010		ND	ND

*Model χ^2^ = 357, df = 10, p<0.0001. ND, not done.  †In multivariate analysis, age groups were merged into 2 groups: <36 y and >36 y.

**Figure 5:**
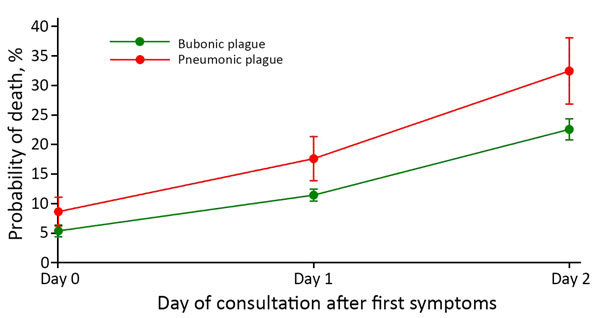
Predictive mortality rate according to clinical form and day of consultation for cases of plague, Madagascar, 1998–2016. Error bars indicate 95% CIs.

Death predictions of the model for BP and PP by day of consultation ranged from 5% to 32%. Predicted CFR of PP increased from 9% (95% CI 6.2%–11.1%) when consulting on first day of symptoms to 32% when consulting after the second day (95% CI 26.8%–38.1%); these values for BP were 5% (95% CI 4.3%–6.4%) and 23% (95% CI 20.8%–24.4%) (Figure 5).

## Discussion

Analysis of 19 years of human plague surveillance in Madagascar showed a distinct peak of confirmed plus presumptive cases during 2003 and 2004. This peak, which might have been influenced by global climate phenomena, such as the El Niño Southern Oscillation and Indian Ocean Dipole (*11**,**12*), was partly caused by an increase in plague cases reported primarily from districts in rural areas of middle-west (including Ambohidratrimo, Miarinarivo, and Tsiroanomandidy) and central (Antananarivo-Renivohitra) Madagascar. The first 3 districts have long been considered plague hotspots (*13*); they reported at least twice the mean confirmed plus presumptive reports during the 5 years before 2004. In 2004, Antananarivo-Renivohitra had a PP outbreak, which was restricted mostly to Arrondissement II of the capital (Antananarivo). This unusual event caused panic among the population and resulted in overreporting, which, combined with misdiagnosis, resulted in a high number of suspected cases that were not confirmed. A similar situation occurred in 1994, when a suspected plague outbreak affected the cities of Surat and Beed in India and caused 54 deaths in 876 reported case-patients (*9*). However, *Y. pestis* was not isolated from this outbreak, and many patients were subsequently given a diagnosis of fatal illnesses resembling acute pneumonia and not plague (*14*).

In our study, total annual cases peaked in 2004 and decreased to the lowest value during 2016 (Figure 2). This reduction occurred primarily in suspected cases and might be caused by improved adherence to case criteria after training and education that occurred during widespread national implementation of the F1RDT starting in 2002.

Monthly distribution of human plague cases in Madagascar in this study was consistent with previous findings, except for a troubling recent trend of the season starting earlier during the year (Figure 3). In general, the human plague season in Madagascar started with early recrudescence in September and peaked in November (*5**,**15*); this pattern is associated with temperature effects on flea abundance and climate and weather patterns (*16*). In more recent years, overall confirmed plus presumptive cases and CFR have both increased in August, during what is typically low season for human plague in Madagascar. Increased CFR in August might have been caused by misdiagnosis by health workers unaccustomed to suspecting plague cases in this month or delayed treatment.

Relatively low specificity (59% overall, 44.7% for PP) of the F1RDT compared with that for culture might be caused by several factors, including badly preserved and contaminated field samples and long delays in transport to laboratory; both factors could lead to negative results from culture even if the cultured material originated from a true plague case. In addition, negative culture results might have resulted from administration of antimicrobial drugs by local health officials before sampling (*17*), whereas F1RDT results remained positive >3 weeks after treatment initiation (*18*). Moreover, pain, fever, and cough are often treated with self-medication in Madagascar (*19*). Thus, specificity of the F1RDT test is almost certainly underestimated by comparing it with culture. Whatever the true rate, F1RDT does lack specificity, which could lead to false-positive results and treatment for persons not truly infected with *Y. pestis*. Fortunately, the sensitivity of F1RDT was 100%, mitigating dangers associated with false-negative results.

Consistent with previous findings from Madagascar during 1957–2001 (*5*), we found that BP occurred most commonly in children and adolescents 5–19 years of age, which might be associated with several factors. First, young children in Madagascar are more likely to be employed in agricultural settings, thus providing increased contact with rodents and fleas (*20*). Second, children sleeping or playing on the ground are exposed to more flea bites (*15*). In Madagascar and other plague-endemic countries (Tanzania, Uganda, and Mozambique), a higher risk for contracting BP is associated with sleeping habits; women and children are at greater risk for exposure to fleas when sleeping directly on the floor or on simple mats (*21**–**23*), and mats are more likely to be infested with fleas than other bedding (*23*).

In contrast, but in agreement with previous findings from Madagascar (*5*), PP was more frequent in adults >30 years of age. High frequency of PP in adults is caused mainly by delayed diagnosis of BP, which then progresses to secondary PP. This delay is associated with multiple behaviors, including self-medication, ignorance of the disease, and trust in traditional healers, which contribute to delay in administration of appropriate treatment by health workers (*2**,**24**–**26*). Also, early clinical symptoms of PP are unspecific, leading to misdiagnosis. PP in adults might also result from participation in funeral ceremonies and attention given to plague patients (*25*).

Death was more common for PP case-patients who had recently traveled to a plague-endemic region (Table 3). Field investigations often found that index case-patients had traveled long distances (50–200 km) between probable places of infection and their home villages (*24**,**25*); this finding was observed in India (*27**,**28*) and the Democratic Republic of the Congo (*29*). Our multivariable model showed that declaring contact with another plague case-patient is a death risk factor after adjustment for PP. This finding could be explained by situations or factors not available in the dataset that are common during major outbreaks and would reduce the survival rate. The model also suggests that lack of observation of dead rats is associated with death. Plague outbreaks are usually associated with observations of dead rats, raising awareness for healthcare providers. Plague case management might be poorer in locations where clinicians are unaware of this indicator.

Spatially, most confirmed plus presumptive cases during 1998–2016 were confined to the Central Highlands, but some occurred in other regions where plague has been historically absent (Figure 4). Concentration of human plague in the Central Highlands, consistent with past trends in Madagascar (*5*), is likely caused by long-term persistence of plague in this region within rodent reservoirs. A recent phylogeographic analysis showed that different and distinct *Y. pestis* subpopulations largely persist at the same geographic locations in the Central Highlands over time, suggesting that plague is constantly and stably maintained locally in rodent populations in this region and not regularly reintroduced from other locations (*13*). During this period, BP cases occurred throughout the Central Highlands, especially in the central and western portions (Figure 4). PP cases were scattered throughout the Central Highlands but concentrated in the northeastern portion in areas that typically report few human plague cases (Figure 4). The relatively high number of PP cases in these areas was caused by misdiagnosis and ignorance of plague by health workers and the local population, which delayed treatment and enabled BP to progress to PP.

Human plague is now occurring in some regions of Madagascar where it was historically absent. An outbreak of BP emerged in southeast Madagascar (Befotaka and Iakora Districts) in November 2016, outside previously known limits of plague-endemic foci. It is not clear whether plague has always been present in these regions and not previously detected or was recently transported to these regions. Imported plague caused by human movements or transportation has been documented for Mahajanga, a coastal location outside the traditional regions of plague in Madagascar, resulting in reintroduction and establishment of plague in this port city. It then cycled in local rodent and flea populations for several years before apparently disappearing (*30*). Because plague can suddenly occur in new locations in Madagascar, clinicians and authorities outside traditional plague foci need to be aware of this risk.

It has been long speculated that plague epidemics in Madagascar occur in 4–5-year cycles; however, no clear periodicity could be defined at the level of rural districts (*15*), which could be attributed to dynamics of plague in rodents and fleas. BP outbreaks are preceded by rodent epizootics (*3*), which reduce local rodent populations; it is unknown how long it takes for rodent populations to recover sufficiently to support another epizootic. Fluctuation in rodent abundance caused by plague might vary depending on species, fecundity, availability of food, or lifespan, or larger factors, such as weather and climate. High abundance of rodents might lead to more contact between humans and animals and, therefore, to outbreaks (*31*). In Himachal Pradesh, India, examination of past plague epidemics showed that outbreaks occur in cycles of 10–15 years (*28*).

Tools for early detection and treatment, as well as properly trained health workers, are critical to reducing overall plague deaths and progression of BP to PP. Just a 2-day delay in treatment dramatically increased death rates for BP and PP (Figure 5); these national findings for PP are similar to those from a single PP plague outbreak in Moramanga in 2015 (*26*). That study highlighted the need for education and rapid clinical decision-making for reducing deaths because the average duration from full onset of PP to death was 1.9 days. Because it is highly sensitive and can be used in remote settings, F1RDT is a proven preliminary diagnosis tool that has led to earlier detection of outbreaks and rapid implementation of control measures in multiple countries in Africa (*32**–**34*). This more local and focused diagnostic capability provides considerable savings in terms of treatment, chemoprophylaxis, and pesticide treatment; in Madagascar, these activities are conducted systematically and free of charge when a plague case is suspected.

An increase in the proportion of PP cases over time (Figure 3) was caused by untreated BP cases progressing to PP and is symptomatic of the deteriorating health system in Madagascar, a result of sociopolitical and economic crises of 2002 and 2009 (*1*). Specifically, this deterioration is likely caused by fast turnover of health workers in plague-endemic regions, especially after changes in the government in Madagascar or political crises; new workers were not always properly trained for plague diagnosis. Surveillance and control activities throughout Madagascar are also affected by lack of financial support. Discontinuation of regular plague surveillance in Antananarivo during 2006–2018, caused by financial shortages, likely contributed to the reappearance of plague in its suburbs 6 years after the previous last reported case. In 2011, two human cases were confirmed there outside the typical season, and *Y. pestis* was also isolated from the spleen of an *R. rattus* rat (*2*). More concerning, in 2017 a large PP outbreak occurred in Madagascar, with many cases in Antananarivo (*35*). A similar overall situation has transpired in the Democratic Republic of the Congo, where civil war and political crisis, leading to a lack of resources, has adversely affected plague control activities (*9*).

Control and prevention of human plague cases in Madagascar faces challenges. BP acquired from a *Y. pestis*–infected flea is the most common form and the ultimate source of PP cases and outbreaks. Thus, prevention strategies are focused on rodent hosts and flea vectors. Unfortunately, inappropriate use of chemical insecticides, contrary to PNCP recommendations, has promoted emergence in Madagascar of fleas resistant to multiple classes of insecticides, leading to survival of *Y. pestis*–infected fleas (*36**,**37*). Human contact with rodent hosts, and consequently flea vectors, is increased by common practices of storing food and disposing of garbage near households (*21*) and close proximity of households in many locations. Fortunately, control of human plague cases is still possible by use of standard antimicrobial drugs (assuming treatment is started in time) because only a few *Y. pestis* strains of >4,000 strains examined have been resistant to major antimicrobial drugs, such as streptomycin and doxycycline (*38**–**40*). Also, plague diagnosis by using F1RDT on dead rodent samples can also contribute to efficient plague prevention (*8*).

This study has major strengths and potential limitations. Strengths include the length of the study and large sample sizes. These sample sizes benefit from the fact that all suspected plague in Madagascar must be reported with standard epidemiologic information. One possible limitation is that diagnostic tools used for case definition evolved during the study. Also, only confirmed plus presumptive cases were analyzed, which might not fully represent the national situation. Furthermore, samples for confirmation were often unavailable from regions where plague had not previously been reported or not reported for several years, a limitation evident for the reemerging focus in Moramanga (*26*) and the recent outbreak in southeastern Madagascar, where many deaths were reported but samples were not always available for confirmation. Thus, risk factors associated with complications were not analyzed for these cases.

In summary, a seasonal pattern of plague was observed in Madagascar during September–March. Annual cases of plague peaked in 2004 and decreased in 2016. This reduction might be caused by improved adherence to case criteria during implementation of the F1 rapid diagnostic test in 2002. Because plague can suddenly occur in new locations in Madagascar, clinicians and authorities outside traditional plague foci need to be aware of this risk.

Appendix Additional information on trends of human plague, Madagascar, 1998–2016.
