# Supplementary material for: Trends of Human Plague, Madagascar, 1998–2016
Source: Emerg Infect Dis. 2019 Feb;25(2):220–8. doi: 10.3201/eid2502.171974 (PMC6346457; doi:10.3201/eid2502.171974)
Supplement: Appendix — Additional information on trends of human plague, Madagascar, 1998–2016. [file 17-1974-Techapp-s1.pdf]

Article DOI: https://doi.org/10.3201/eid2502.171974

Trends of Human Plague, Madagascar, 1998–2016

Appendix

Human Plague Surveillance

In Madagascar, all clinically suspected plague cases that meet clinical and epidemiologic criteria specified in the World Health Organization standard case definition are reported to the national surveillance system. All data analyzed here were gathered from this system during 1998–2016; no ethics approval was required. Declaration forms of all suspected cases were compiled at the Central Laboratory for Plague (CLP) of the Malagasy Ministry of Health.

The information form was divided into 4 parts. Part 1 included information related to the affected area (place). Part 2 included details about the suspected patient (person), clinical information (presence of bubo [painful or not, size and location], and any pulmonary signs (cough >5 days, bloody sputum or chest pain); patient status (alive or dead); and disease onset (time). Part 3 included epidemiologic information indicating plague circulation among local rodent populations (recorded as rodent deaths over the past 15 days). Part 4 included information about contacts of the patient before infection (other plague patient or deaths among the family and neighbors of the patient) and information on possible sources of the infection and epidemiologic links. A reference number was assigned to each reported plague case-patient and then reported on contact reference number of other reported case-patients having links with the case-patient, enabling detection of infection clusters: groups of ≥2 confirmed or presumptive case-patients who declared having physical contact, being in close proximity, or some other possible infection link with each other. Active plague foci were followed-up daily for 12 days after the last case (twice the plague infection incubation period). If no case was reported during this period, then the outbreak was considered complete and was defined as 1 distinct plague episode.

For each suspected plague patient, heat-fixed smears and biologic samples (e.g., bubo aspirates, sputum, or postmortem organ punctures) were mailed to the CLP. Microscopy for

smears was performed until 2001 as a presumptive test but was discontinued after the F1 Rapid Diagnostic Test (*1*) was routinely used as a presumptive test (Figure 1); this test was distributed to all districts and other healthcare centers in plague-endemic areas of Madagascar by 2002 for rapid diagnosis at the patient bedside. Samples collected on sterile swabs were conveyed in Cary-Blair transport medium for confirmation by culture at the CLP.

Reference

1. Chanteau S, Rahalison L, Ralafiarisoa L, Foulon J, Ratsitorahina M, Ratsifasoamanana L, et al. Development and testing of a rapid diagnostic test for bubonic and pneumonic plague. Lancet. 2003;361:211–6. PubMed http://dx.doi.org/10.1016/S0140-6736(03)12270-2
